# Selection of Metastatic Breast Cancer Cells Based on Adaptability of Their Metabolic State

**DOI:** 10.1371/journal.pone.0036510

**Published:** 2012-05-03

**Authors:** Balraj Singh, Karen Tai, Simran Madan, Milan R. Raythatha, Amanda M. Cady, Megan Braunlin, LaTashia R. Irving, Ankur Bajaj, Anthony Lucci

**Affiliations:** Department of Surgical Oncology, The University of Texas MD Anderson Cancer Center, Houston, Texas, United States of America; University of Navarra, Spain

## Abstract

A small subpopulation of highly adaptable breast cancer cells within a vastly heterogeneous population drives cancer metastasis. Here we describe a function-based strategy for selecting rare cancer cells that are highly adaptable and drive malignancy. Although cancer cells are dependent on certain nutrients, e.g., glucose and glutamine, we hypothesized that the adaptable cancer cells that drive malignancy must possess an adaptable metabolic state and that such cells could be identified using a robust selection strategy. As expected, more than 99.99% of cells died upon glutamine withdrawal from the aggressive breast cancer cell line SUM149. The rare cells that survived and proliferated without glutamine were highly adaptable, as judged by additional robust adaptability assays involving prolonged cell culture without glucose or serum. We were successful in isolating rare metabolically plastic glutamine-independent (Gln-ind) variants from several aggressive breast cancer cell lines that we tested. The Gln-ind cells overexpressed cyclooxygenase-2, an indicator of tumor aggressiveness, and they were able to adjust their glutaminase level to suit glutamine availability. The Gln-ind cells were anchorage-independent, resistant to chemotherapeutic drugs doxorubicin and paclitaxel, and resistant to a high concentration of a COX-2 inhibitor celecoxib. The number of cells being able to adapt to non-availability of glutamine increased upon prior selection of cells for resistance to chemotherapy drugs or resistance to celecoxib, further supporting a linkage between cellular adaptability and therapeutic resistance. Gln-ind cells showed indications of oxidative stress, and they produced cadherin11 and vimentin, indicators of mesenchymal phenotype. Gln-ind cells were more tumorigenic and more metastatic in nude mice than the parental cell line as judged by incidence and time of occurrence. As we decreased the number of cancer cells in xenografts, lung metastasis and then primary tumor growth was impaired in mice injected with parental cell line, but not in mice injected with Gln-ind cells.

## Introduction

The success of cancer depends on ongoing generation of genetic and epigenetic heterogeneity in cancer cells, combined with the selection of cells that are capable of adapting and surviving numerous obstacles in the body [1]–[6]. The process of metastasis, requiring multiple rate-limiting steps, is very inefficient. Less than 0.01% of cancer cells within the blood circulation survive to produce experimental lung metastasis [5]–[7]. Cancer cells present in metastases are also heterogeneous, only a small subpopulation of them being able to produce new metastasis in patients or in experimental models [5]. Tumor heterogeneity can be explained by 1) developmental hierarchy wherein a small percentage of cells are stem cell-like thus giving rise to progeny of different characteristics than the mother cell [6], and/or 2) high genomic instability of metastatic cancer cells [8], [9]. In breast cancer, indeed in most cancers, clinical metastasis is an end result of a long Darwinian evolution-like selection process spanning decades, in which the most adaptable cancer cells persist. The selection pressures in the body include metabolic challenges, and attacks from the immune system. To develop effective therapeutic strategies against metastatic disease, it would be desirable to use *in vitro* systems that contain cellular heterogeneity and high adaptability similar to what is present in clinical metastases.

*In vitro* selection approaches are ideally suited for selecting cell variants that can survive metabolic challenges. Numerous studies deal with investigating how oncogenes cause dysregulation of metabolism, particularly aerobic glycolysis, which increases glucose utilization in cancer cells [10]. Our approach is different in that we decided to utilize the adaptability of metabolic state for selecting metastatic cancer cells from a heterogeneous population. One important reason behind our approach is that disseminated tumor cells present in the bone marrow of stage I–III breast cancer patients survive for years in a relatively dormant state before causing clinical metastasis [11], [12]. So the cancer cells that are ultimately responsible for clinical metastasis should be able to survive metabolic challenges imposed for prolonged periods.

Glutamine (Gln) has important roles in cancer cells in addition to being a building block in proteins, including being a source of carbon and nitrogen for anabolic pathways [13]. Glutamine is also essential for the uptake of essential amino acids, which involves simultaneous efflux of Gln using a bidirectional transporter [14]. In this study, we investigated whether there are variant cells present in aggressive breast cancer cell lines that can survive lack of Gln, and whether such cells are adaptable and therefore more tumorigenic and metastatic than the parental cell line. Dependence of cancer cells on exogenous Gln is driven by Myc protein, which suppresses miR-23a and miR-23b, leading to the induction of glutaminase (GLS) [15]. Myc protein, which is frequently overexpressed in aggressive breast cancers, is a key regulator of metabolism [16] and it is also well known for driving poor-prognosis gene expression signatures and an embryo-like phenotype [17], [18].

COX-2 plays a key role in breast cancer metastasis as revealed by studies with COX-2 overexpressing breast cancer cell lines, a xenograft mouse model of bone metastasis, and the use of a COX-2 inhibitor in a bone metastasis model [19]. The molecules whose expression correlates with COX-2 overexpression in metastatic clones include well known NFκB targets urokinase plasminogen activator and interleukin-8 that are involved in invasion and angiogenesis [20], [21]. Others have provided convincing evidence for the involvement of COX-2 in breast cancer metastasis to lung and to brain [22], [23]. Using a commonly used sphere culture method developed in another laboratory, we found that COX-2 has an important role in cancer stem-like cells [24]. COX-2 overexpression promotes genomic instability and also leads to therapeutic resistance [25], [26]. Aggressive breast cancers, including inflammatory breast cancers (IBC), overexpress a number of NFκB target genes. In an analysis of 60 NFκB target genes, 35 were found to be overexpressed in primary tumors in IBC [27]. Interestingly, COX-2 and CXCL1 were the only two genes of the 35 that are also co-expressed in metastases [27]. Importantly, COX-2 expression in the primary tumor of stage I-III breast cancer patients predicts tumor cells dissemination to bone marrow [28], which is known to predict metastasis in the future [11]. Significantly, NFκB inhibition in SUM149 IBC cell line inhibits metastasis in nude mice [29]. With this overwhelming evidence in support of COX-2 involvement in breast cancer metastasis, we chose to analyze COX-2 overexpression as an indicator of metastatic potential of *in vitro* selected metabolically plastic cancer cells, prior to xenograft experiment in nude mice. We also investigated using siRNA-mediated knockdown how COX-2 signals differently in Gln-ind cells as compared to other COX-2 overexpressing cells.

## Results

### Selection of Gln-ind variants from aggressive Gln-addicted breast cancer cell lines

**Figure 1:**
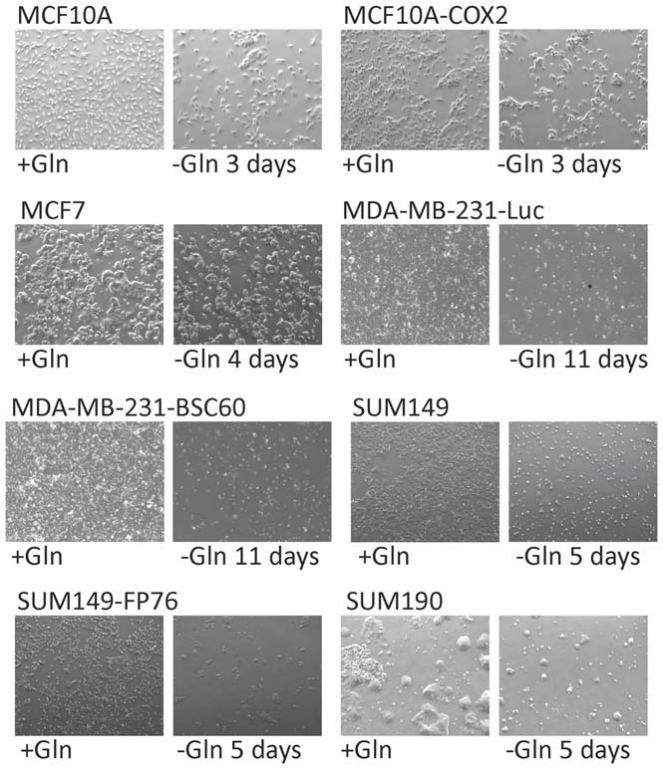
Effect of glutamine deprivation on breast cancer cell lines. Indicated cell lines were cultured with or without Gln as described in Materials and Methods. Photographs of cultures in complete medium (+Gln) versus in Gln-free medium (−Gln) are shown. Results are summarized in Table 1.

**Figure 2:**
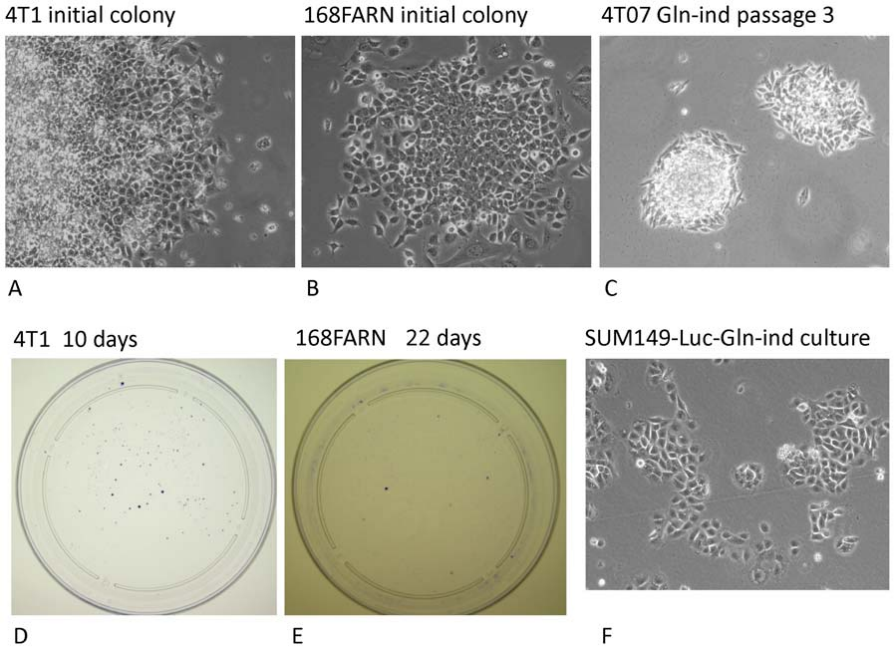
Selection of Gln-ind variants from breast cancer cell lines. (A–C) Half a million cells were plated in a 10-cm dish. The next day, the medium was changed to a Gln-free medium containing dialyzed fetal bovine serum. Colonies of cells growing under these conditions for 2–4 weeks were photographed. (D, E) Dishes of colonies stained with crystal violet. (F) SUM149-Luc-Gln-ind cells growing in Gln-free medium.

We began by investigating the dependence of breast cancer cell lines on Gln in culture medium. Our approach was to determine the subpopulation of cells that would survive long-term. We observed that Gln withdrawal did not appreciably inhibit the growth of normal MCF10A, pre-malignant MCF10A-COX2, or poorly metastatic MCF7 cell lines (Fig. 1). There was approximately 50% decrease in cell number in all 3 cases as compared to control in 4 days, but then surviving cells grew to confluency and could be cultured long term. In contrast, Gln withdrawal severely inhibited the metastatic cell lines MDA-MB-231, SUM149, and 4T1 (>99% of cells died in all 3 cell lines). These results indicate that breast cancer cell lines are addicted to Gln, and the degree of addiction correlates with metastatic ability of cell lines. We followed luciferase-transfected SUM149-Luc cells in culture medium lacking Gln to determine whether some rare cells would survive several weeks without Gln. More than 99.99% cells died under these conditions. However, we were surprised to find that a few cells not only survived but resumed proliferation in Gln-free medium. We obtained 13 colonies of such cells that were easily visible to the naked eye in four weeks in a representative dish that originally contained a half million cells. Therefore, the proportion of cells that survived and proliferated was 0.01% or less. We were able to culture these Gln-independent (Gln-ind) variant cells in a Gln-free medium for multiple passages (>16). We were able to similarly select rare Gln-ind cells from most breast cancer cell lines, including some very aggressive breast cancer cell lines in addition to SUM149, although they differed in properties (Fig. 2, Table 1). For example, rare Gln-ind colonies selected from the following cell lines could be cultured long-term in glutamine-free medium: SUM149-FP (cell line established in our laboratory from a fat pad tumor), SUM190 IBC cell line, 4T07, and 168FARN (both mouse breast cancer cell lines). On the other hand, although some rare cells survived in MDA-MB-231 and MDA-MB-231-BSC60 (metastatic clone) human and 4TI mouse breast cancer cell lines, they required Gln for long-term proliferation. Significantly, we were able to culture Gln-ind cells from freshly isolated fat pad tumors from the nude mice that were inoculated with either parental SUM149 cell line or its *in vitro* selected Gln-ind variants (B Singh, S Madan, and A Lucci, unpublished observations), further emphasizing the relevance of such cells in tumor formation.

**Table 1:** Glutamine-dependence of breast cancer cell lines.

Cell line	Properties	Growth inhibition in Gln-free medium
MCF10A	Normal	Weak inhibition, grew to confluency
MCF10A-COX2	Premalignant	Weak inhibition, grew to confluency
MCF7	Poorly metastatic	Weak inhibition, grew to confluency
MDA-MB-231	Metastatic	Severe inhibition, but variants survived
BSC60	Highly metastatic, bone-seeking clone	Severe inhibition, but variants survived
SUM149	Metastatic and locally aggressive	Severe inhibition, but variants grew long term
SUM149-Luc	Metastatic and locally aggressive	Severe inhibition, but variants grew long term
SUM149-Luc-FP	Cultured from a fat pad xenograft	Severe inhibition, but variants grew long term
SUM190	Metastatic and locally aggressive	Severe inhibition, but variants grew long term
4T1	Highly metastatic	Severe inhibition, but variants yielded colonies
4T07	Forms micrometastases in lungs	Severe inhibition, but variants grew long term
168FARN	Yields lymph node metastases	Severe inhibition, but variants grew long term

BSC60, metastatic clone derived from MDA-MB-231.

Luc, luciferase transfected cell line.

### Molecular basis of metabolic plasticity of Gln-ind cells

**Figure 3:**
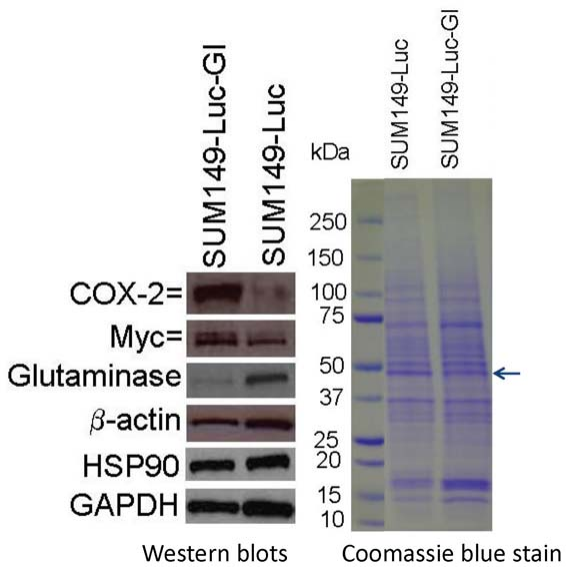
Low glutaminase level in Gln-ind cells. Selected proteins likely to be involved in Gln addiction (GLS, Myc) and in metastasis (COX-2, Myc) were analyzed by western blotting in Gln-ind and parental SUM149-Luc cells. Since the β-actin level was found to be lower in the Gln-ind cells than in the parental cell line by western blotting, we included additional gel loading controls: western blotting with Hsp90 and GAPDH antibodies, and Coomassie blue staining of a gel run in parallel.

To first address how Gln-ind cells survived lack of Gln, we determined whether they had a reduced level of GLS (which converts Gln to Glu) than the parental cell line. Our western blot analysis showed that the Gln-ind variants had a GLS level less than 20% that in parental SUM149 cells (Fig. 3). We interpret that the absence of exogenous Gln makes it necessary that the proliferating Gln-ind cells synthesize and preserve sufficient Gln needed for essential functions (e.g., protein synthesis), and therefore reduce their GLS level. In addition, metabolic enzymes including GLS are well known for their allosteric regulation to suit intracellular levels of substrates and products; such regulation is also likely to occur under lack of exogenous Gln. Next, we determined whether the key regulator of GLS, i.e., Myc protein, was also down-regulated in Gln-ind cells. Significantly, we found that the reduction in GLS level in Gln-ind cells was not accompanied by a reduction in Myc level. On the contrary, Gln-ind cells contained a modestly elevated level of Myc protein (50% more than the parental cell line; Fig. 3).

We noted that β-actin level was lower in the Gln-ind cells than in the parental cell line, both by western blotting and by Coomassie blue staining (actin-sized band is indicated by an arrow in Fig. 3). Therefore, we used additional controls (Hsp90 and GAPDH) for normalizing gel loading. Protein levels of GAPDH and some additional glycolytic enzymes (not shown) were also lower in Gln-ind cells than the parental cell line, which could be a part of the overall slow rate of glycolysis in the absence of glutamine.

### Overexpression of COX-2 in Gln-ind cells

**Figure 4:**
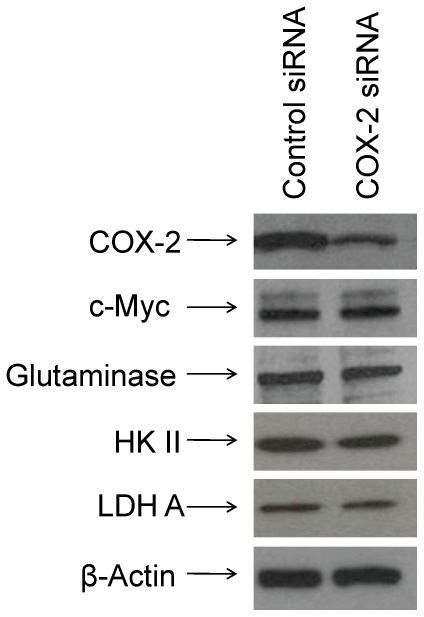
COX-2 knockdown does not affect Myc, GLS, HK II, and LDH A protein levels in Gln-ind cells. We transfected SUM149-Gln-ind cells growing without Gln with a COX-2-specific siRNA or with a control siRNA and analyzed the levels of Myc and some proteins involved in Myc-mediated glycolysis (hexokinase II and lactate dehydrogenase A) and glutaminolysis (glutaminase) by western blotting compared with those in the parental SUM149-Luc cell line.

**Figure 5:**
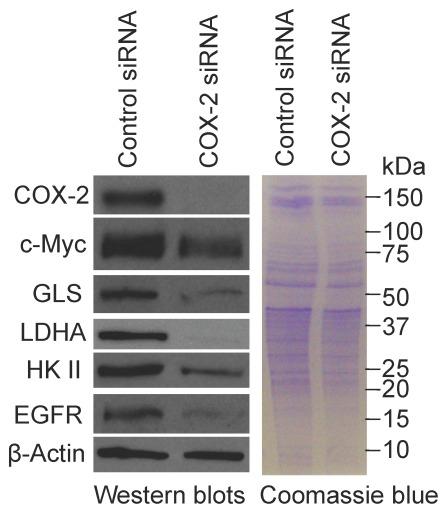
COX-2 function is linked with Myc in regulating glycolysis and glutaminolysis in the COX-2-overexpressing, celecoxib-resistant SUM149-CER cell line. We transfected SUM149-CER cells with a COX-2-specific siRNA or with a control siRNA and analyzed the levels of Myc and some proteins involved in Myc-mediated glycolysis (hexokinase II and lactate dehydrogenase A) and glutaminolysis (glutaminase) by western blotting.

**Figure 6:**
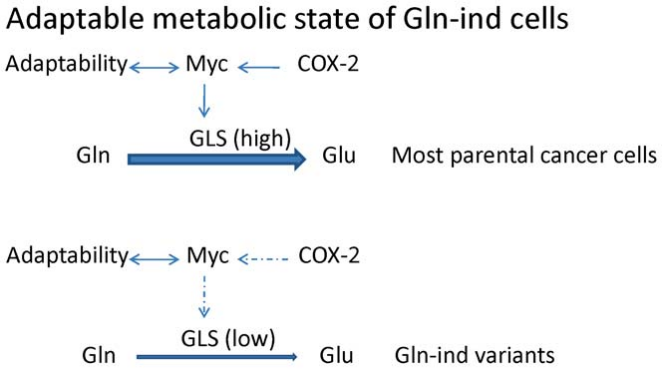
Model summarizing western blot analysis and additional data. Adaptable Gln-ind cells have high levels of COX-2 and Myc proteins, but they are able to dissociate Myc expression and GLS expression. The majority of cells lacking such adaptability may have perished in the initial selection in Gln-free medium. Broken arrows indicate adaptability to dissociate COX-2 function from Myc function, and Myc function from GLS level in Gln-ind cells, as identified in this study.

To determine whether Gln-ind cells have a higher malignancy potential than the parental cell line, we first analyzed COX-2 protein level. We found by western blotting that Gln-ind cells produce approximately 5-fold higher level of COX-2 protein than the parental cell line (Fig. 3). Next, we determined whether there is a close linkage between increased COX-2 level and decreased GLS level in Gln-ind cells. We found that siRNA-mediated knockdown of COX-2 did not alter GLS level in Gln-ind cells indicating that a high COX-2 expression is not directly linked with a reduced GLS level (Fig. 4). This result prompted us to further investigate the relationship of COX-2 signaling with the synthesis of metabolic enzymes that are regulated by Myc protein, including GLS, hexokinase II, and lactate dehydrogenase A (LDHA). We knocked down COX-2 by siRNA both in Gln-ind cells that overexpress COX-2 and in celecoxib-resistant SUM149-CER cells that overexpress COX-2 [30], to determine whether COX-2 signals differently in metabolically plastic cells as compared to other cells. On the basis of the number of cells that survive selection without Gln (<0.01%) versus the cells that survive celecoxib treatment (1–10%), Gln-ind cells may be a small subpopulation of COX-2^high^ cells present in SUM149 cell line that survive in the presence of celecoxib. COX-2 knockdown in SUM149-CER cells, but not in Gln-ind cells, resulted in reduced levels of Myc protein and the metabolic enzymes Myc regulates: GLS, hexokinase II, and LDHA (compare Figs. 4 and 5). We interpret that signaling nodes in Gln-ind cells, e.g., COX-2 and Myc, communicate with metabolic state in a highly adaptive manner (see model in Fig. 6). Gln-ind cells can lower the GLS level in response to the non-availability of exogenous Gln even when Myc and COX-2 levels are high.

We also analyzed the effect of COX-2 knockdown in SUM149-CER cells on epidermal growth factor receptor (EGFR) level by western blotting. EGFR plays a major role in aggressive breast cancers, including IBC. EGFR overexpression is also important in the survival of breast cancer cells under low-glucose culture conditions, this effect being independent of its protein kinase activity [31]. Interestingly, we found that COX-2 knockdown resulted in a significant reduction in EGFR level in SUM149-CER cells (Fig. 5) suggesting a crosstalk between COX-2 signaling and EGFR expression.

### Mechanism of COX-2 overexpression

**Figure 7:**
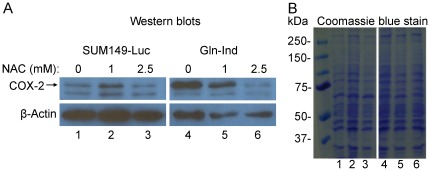
A reduction in COX-2 level upon treatment with N-acetyl cysteine. We exposed parental SUM149-Luc and Gln-ind cell lines, both growing in a medium with glutamine, to N-acetyl cysteine for 24 hours before subjecting them to western blotting using equal volumes of cell lysates. Since β-actin was not a reliable indicator of protein loading, we ran a gel in parallel and stained it with Coomassie blue. Lane numbers 1–6 at the bottom in left panel correspond to lane numbers 1–6 in the right panel.

A common trigger for COX-2 overexpression is oxidative stress. To determine whether oxidative stress may be driving COX-2 overexpression in Gln-ind cells, we analyzed the intracellular level of reduced glutathione (GSH), an indicator of redox status. We found that Gln-ind cells have a lower level of GSH than the parental SUM149-Luc parental cell line, thus supporting our hypothesis (Fig. S1). Because of an extremely low level of reduced glutathione in Gln-ind cells, which is close to lower limit of detection in our assay, we were not able to accurately determine percentage of reduction. We also found that Gln-ind cells have a low level of oxidized glutathione (GSSG) as well (approximately 40% of the level in parental cell line; Fig. S1), indicating that they have a low capacity to regulate redox status. In an alternative approach, we found that addition of N-acetyl cysteine (NAC) in culture medium for 24 hours to lower oxidative stress resulted in a lower COX-2 production in Gln-ind cells (Fig. 7). At 1 mM dose NAC inhibited COX-2 production in Gln-ind cells, but not in parental SUM149-Luc cells (Fig. 7). At a higher 2.5 mM dose NAC inhibited COX-2 in both Gln-ind and parental cell line, although percent inhibition was higher in Gln-ind cells. Stress mediated induction of COX-2 may involve NFκB transcription factor binding to COX-2 gene promoter, although other mechanisms are also possible. To determine whether NFκB is involved, we added a specific inhibitor of NFκB activation pathway, BAY-11-7082, to the culture medium for 24 hr. Analysis of COX-2 by western blotting showed that BAY-11-7082 treatment caused a decrease in COX-2 level in Gln-ind cells growing with or without Gln (Fig. S2). COX-2 level in the parental cells remained unaffected under these conditions (Fig. S2).

### Adaptability in Gln-ind cells

**Figure 8:**
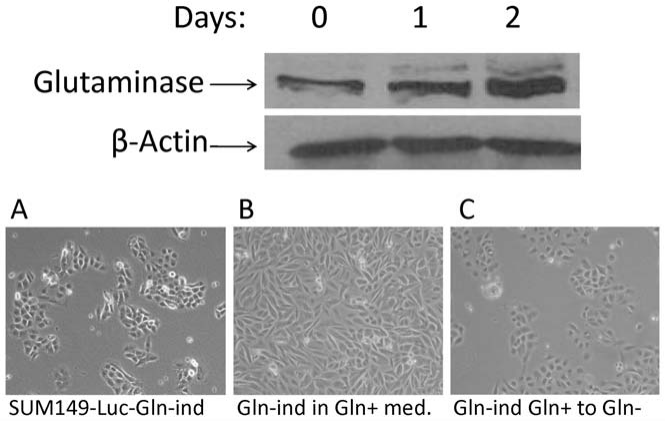
Glutamine-independent phenotype is adaptable but stable. **Top panel:** Induction of GLS upon growth in glutamine-containing medium. SUM149-Gln-ind cells growing in Gln-free medium were shifted to Gln-containing medium, and GLS was analyzed by western blotting at different time points after the medium change. **Bottom panel:** Morphologies of cells growing under different conditions are shown. (A) Gln-ind cells growing without Gln. (B) Gln-ind cells growing in the presence of Gln (they exhibited faster growth and altered morphology upon growth with Gln). (C) Gln-ind cells were maintained in Gln-containing medium for 8 passages and then switched to Gln-free medium for 7 days.

We found that Gln-ind cells can utilize Gln as efficiently as the parental cell line. Within a day after adding Gln to the culture medium, we observed an induction of GLS (Fig. 8 top). The Gln-ind cells proliferated approximately 50% slower in the Gln-free medium than the parental cell line proliferated in the Gln-containing medium. However, upon the addition of Gln, Gln-ind cells resumed a proliferation rate similar to that of the parental cell line. These results indicate that the rare cells that survive the lack of Gln are not defective in Gln utilization; instead, they possess a more adaptable metabolic state. After culture in Gln-containing medium for 8 passages, Gln-ind cells maintained the ability to proliferate without Gln. Gln withdrawal slowed their growth, as expected, but most cells survived and resumed proliferation (Fig. 8 bottom). These results indicate that Gln-ind cells maintain an adaptable metabolic state in Gln-containing medium.

**Figure 9:**
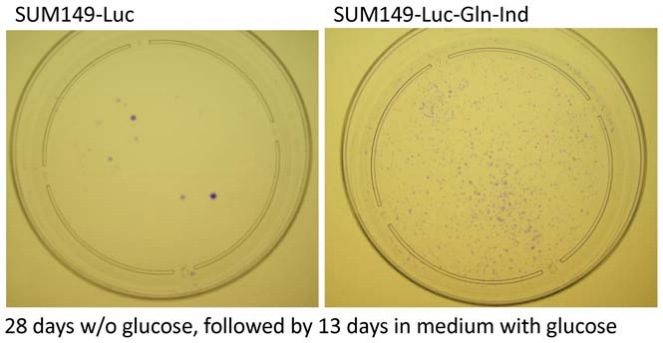
Higher adaptability/survival of Gln-ind versus parental cells in the absence of glucose. Cell cultures were deprived of glucose for 28 days and then allowed to recover and grow for 13 days before crystal violet staining of colonies. Gln-ind cells (right) yielded a large number of colonies, although the colonies were of smaller size.

Next, we determined whether the SUM149-Gln-ind variants are adaptable under other metabolic challenges, e.g., a non-availability of glucose. We cultured cells in glucose-free medium for 28 days (the medium included Gln for both cell lines) and then replaced the medium with complete medium containing glucose. To determine the number of (clonogenic) cells that survived the lack of glucose, we stained the dishes with crystal violet after 13 days in complete medium. The Gln-ind variants survived lack of glucose better than the parental cell line did, as judged by the number of colonies (Fig. 9). One way to explain this result is that non-availability of Gln forces a selection of cells that can slow their overall metabolism, including glucose utilization. In support of this idea, we had noted that Gln-ind cells produced approximately 50% lower level of a glycolytic enzyme glyceraldehyde 3-phosphate dehydrogenase as compared to the parental cell line (Fig. 3). Since glucose has major roles in cellular metabolism, and also in governing the metabolic state [32]–[34], the ability of Gln-ind cells to survive without glucose implies that they are highly adaptable.

### Survival and growth of Gln-ind cells without serum

**Figure 10:**
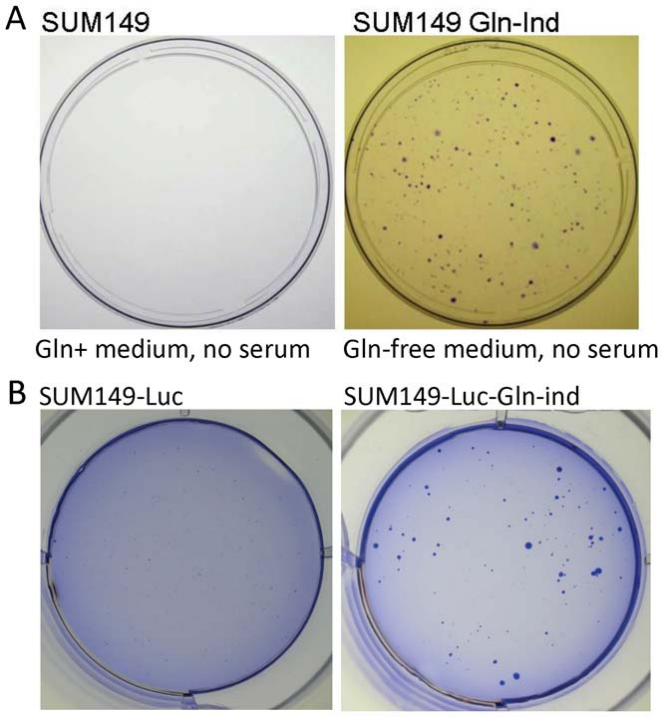
(A) Higher adaptability/survival of Gln-ind versus parental cells in the absence of serum. Cell cultures were deprived of serum for 25 days, and resulting colonies were stained with crystal violet. (B) Higher anchorage-independence of Gln-ind versus parental cells. Twelve thousand cells were cultured in Gln-containing medium with 0.35% low-melt agarose for 23 days and the resulting colonies were stained with crystal violet.

Over the course of the disease, metastatic cancer cells must be able to survive not only metabolic challenges, but also a lack of growth factors. Therefore, it may be useful to know whether Gln-ind cells are more capable of surviving without growth factors than the parental cell line. We have reported previously that COX-2 overexpression in MCF10A cell line can reduce the requirement of EGF in the culture medium for cell growth [25]. We analyzed the effect of withdrawing serum from the culture medium for an extended period (25 days). We found that some Gln-ind cells survived and proliferated, yielding large colonies, while the parental cell line did not yield any such colonies (Fig. 10A). This result suggests that the Gln-ind cells are less dependent on growth factors for their survival and growth. This property could be potentially advantageous in the process of tumor growth and metastasis by reducing a dependence on stroma/microenvironment.

### Gln-ind cells are anchorage-independent and resistant to doxorubicin and paclitaxel

**Figure 11:**
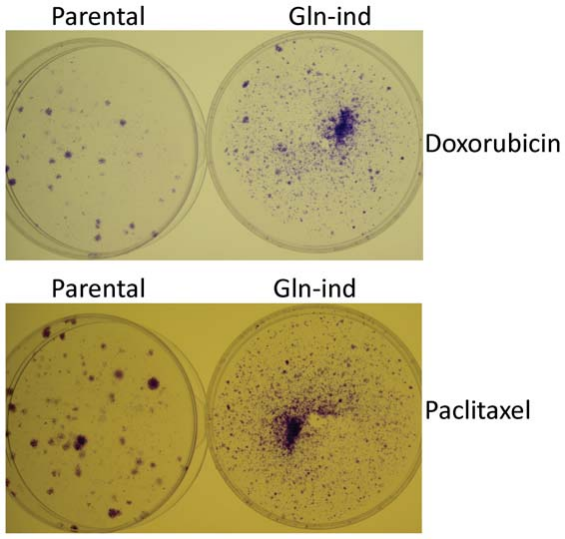
Increased resistance to doxorubicin and paclitaxel in Gln-ind cells. Parental SUM149-Luc cell line or Gln-ind cells were treated with 200 nM doxorubicin (top) or 5 nM paclitaxel (bottom) for 7 days as described in Materials and Methods, and allowed to recover in a drug-free medium for 2 weeks (doxorubicin-treated) or 1 week (paclitaxel treated) before staining the colonies.

Next, we performed some *in vitro* assays to determine whether the Gln-ind cells were more aggressive than the parental cell line. First, in soft-agar assay to determine their anchorage-independent growth, the Gln-ind cells yielded more and much larger colonies than the parental cell line (Fig. 10B). Then we compared Gln-ind and parental cells for their ability to survive in presence of commonly used chemotherapeutic drugs doxorubicin and paclitaxel. The cells were exposed to 200 nM doxorubicin or 5 nM paclitaxel for 7 days in a medium containing glutamine. Approximately 99% cells died under this treatment. To determine the relative survival of resistant cells, we removed the drug by changing the culture medium with the fresh medium without drug, and allowed time for colonies to grow before staining them. In this manner we found that Gln-ind cells yielded more colonies than parental cells under both doxorubicin and paclitaxel treatments (Fig. 11). Thus, a selection for an adaptable metabolic state in cancer cells also selects for chemotherapy resistance. As a complimentary approach to show a relationship between Gln-ind phenotype and chemotherapeutic resistance, we found that SUM149 cells first selected to survive several days treatment with doxorubicin or paclitaxel had an increased survival upon Gln withdrawal than the unselected cells, as judged by the number of colonies (Fig. S3). In such experiments, the chemoresistant surviving cells were first allowed to recover in a drug-free complete medium prior to subjecting them to glutamine withdrawal for 4–5 weeks. Previous studies with COX-2 overexpressing breast cancer cells and chemotherapy resistant breast cancer cells suggest that the high COX-2 expression in Gln-ind cells may account for their resistance to chemotherapeutic drugs [26], [30].

### COX-2 overexpression in Gln-ind cells correlates with celecoxib resistance

**Figure 12:**
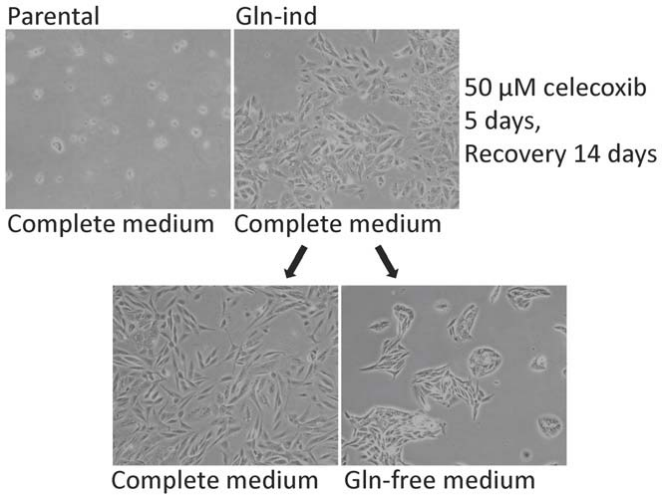
Celecoxib resistance in Gln-ind cells. One million cells of parental SUM149-Luc cell line or Gln-ind cells were treated with 50 µM celecoxib and rare surviving cells were allowed to grow into colonies. Then cells were dispersed by trypsinization and plated in dishes in medium with glutamine (complete medium) or without glutamine.

COX-2 expression in breast cancer cells may correlate with response to a COX-2 inhibitor, e.g., celecoxib. But COX-2 overexpression may also be responsible for resistance to celecoxib in a subpopulation of breast cancer cells [30], [35], [36]. We have found that a small subpopulation of SUM149 cells can tolerate 10–20 µM celecoxib [30]. Celecoxib-resistant SUM149 cells express a higher level of COX-2 than the parental cell line, indicating that the resistance to celecoxib is driven by COX-2 overexpression [30]. In another studies involving mouse models of breast cancer, a COX-2 inhibitor (celecoxib or SC-236) delayed/inhibited tumor growth; however, resistance to COX-2 inhibitors developed due to overexpression of COX-2 in tumors [35], [36]. Initially we found that both parental cell line and Gln-ind cell line contain a significant number of cells that readily survive exposure to 10 µM celecoxib. To determine whether Gln-ind cells have a high potential to drive resistance to celecoxib, we treated Gln-ind cells and parental SUM149-Luc cell line (both in complete medium with glutamine) with 50 µM celecoxib for 5 days. Then we allowed the celecoxib-resistant cells to recover in the medium without celecoxib. In this manner we found that Gln-ind cells, but not parental cell line, contained cells capable of surviving in the presence of 50 µM celecoxib for 5 days. Such celecoxib-resistant Gln-ind cells produced colonies of cells that could be grown indefinitely in culture (Fig. 12).

To further demonstrate the importance of COX-2 in the Gln-ind phenotype, we first selected cells from the parental cell line after treatment with 10 µM celecoxib for 7 days, allowed them to recover in a drug-free medium for 3 days, and then subjected them to Gln withdrawal. In this manner we found that celecoxib-resistant SUM149 cells produced more Gln-ind colonies than the parental cell line treated with the solvent DMSO alone (Fig. S4). These results indicate that COX-2 overexpression has an important role in the survival of Gln-ind cells in the absence of Gln.

### Gln-ind cells are more tumorigenic and more metastatic than the parental cell line

We determined the ability of Gln-ind cells to form tumors and to metastasize in a nude mouse xenograft model. We injected 200 to 2 million luciferase-transfected cells into the thoracic fat pads of nude mice in duplicate and monitored tumor growth and skin metastasis by using whole-body luciferase imaging. Because of a relatively high luciferase signal emanating from the primary tumor in fat pad, which is in the vicinity of lungs, it is not feasible to detect lung metastasis by luciferase imaging. Therefore, we chose to analyze lung metastasis by performing a luciferase activity assay in homogenates from lungs. We showed in a recent study that when nude mice were injected into fat pad with 2 million SUM149-Luc cells, all mice developed lung metastasis as detected by luciferase activity [37]. This result is consistent with an earlier study from another laboratory in which 1 million SUM149 cells were injected and lung metastases were analyzed by examining foci [29]. In another study performed with our SUM149-Luc cell line, lung metastasis was detected with *ex vivo* luciferase imaging (inject D-luciferin into mice, harvest lungs, and image), which correlated with detection by hematoxylin and eosin staining of a lung tissue section [38].

**Figure 13:**
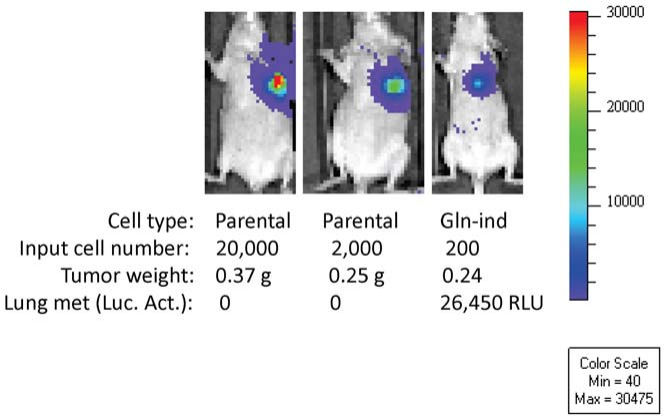
Increased lung metastasis in nude mice injected with Gln-ind cells. Luciferase images of mice injected into fat pad with indicated cell type and cell number were collected at day 83. The three mice were sacrificed on day 90, their primary tumors were weighed, and luciferase activity was determined in their lungs. The data indicate that lung metastasis was impaired in the mice injected with 20,000 or 2,000 SUM149-Luc cells. In contrast all mice injected with Gln-ind cells, including those injected with 200 cells, developed lung metastasis.

**Figure 14:**
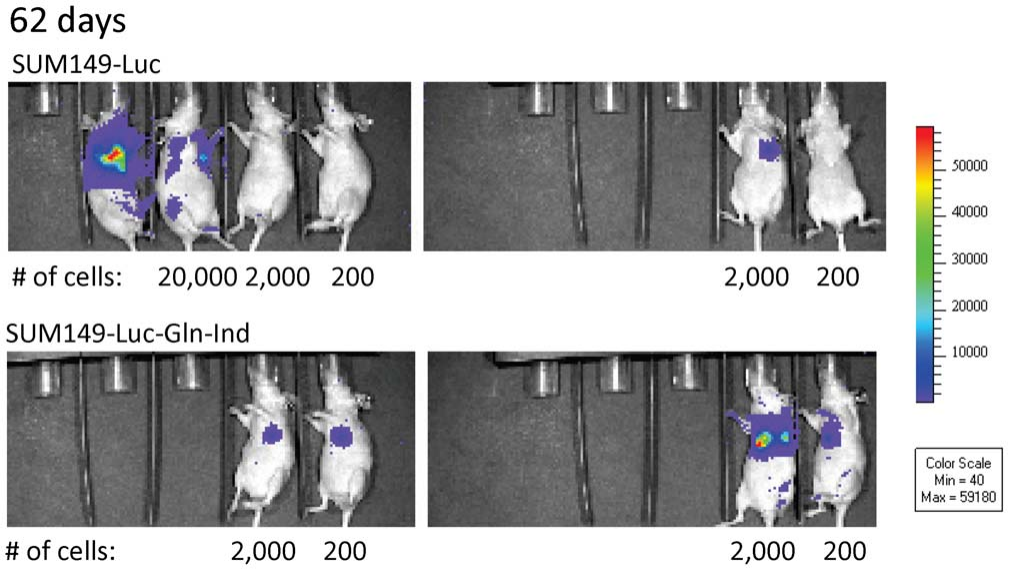
Impairment of primary tumor growth in mice injected with parental SUM149-Luc cells, but not with Gln-ind cells, upon reducing the number of injected cells. Cells (200 to 2 million) were injected into thoracic fat pads of 44-day-old nude mice (decreasing cell number from left to right) in duplicate. The luciferase images collected at day 62 show that primary tumor growth in the mice injected with the parental cell line was significantly reduced or absent upon reducing the number of injected cells below 2,000. In contrast, all mice injected with Gln-ind cells developed tumors, including both mice injected with only 200 cells. Empty slots on the left correspond to the mice that needed to be sacrificed because of high tumor burden.

In the present study, mice injected with 2 million or 200,000 SUM149-Luc parental cells developed lung metastases. However, lung metastases were detected in only one of the two mice injected with 20, 000 SUM149-Luc cells (both mice developed primary tumor). Lung metastasis was not detected in the one mouse injected with 2,000 SUM149-Luc cells that developed primary tumor (the second mouse failed to develop primary tumor or lung metastasis). In contrast, all the mice injected with Gln-ind cells, including those injected with 200 cells, developed lung metastases. Figure 13 includes luciferase images of 2 mice in the control group and one mouse in the Gln-ind group, their tumor weights, and luciferase activity in lungs; all the data shown in Figure 13 was collected in parallel at the same time. Significantly, as the number of injected cells was reduced, lung metastasis was not observed in the mice injected with the parental cell line even though they had good primary tumor growth (Fig. 13). We sacrificed all the remaining mice in this experiment at day 153 after injecting the cells. We found that one mouse injected with 200 parental SUM149-Luc cells developed a very slow growing tumor, detectable by luciferase imaging after 3 months and primary tumor weighing 0.04 g at day 153. The other mouse injected with 200 parental SUM149 cells and one of the two mice injected with 2,000 parental SUM149 cells lacked tumor growth by day 153. All mice injected with Gln-ind cells, including both mice injected with only 200 cells, developed tumors that could be easily observed by luciferase imaging as early as 62 days (Fig. 14). Relative tumor burden in all remaining mice at an early time point, as detected by luciferase imaging at day 34, is also shown (Fig. S5). To sum up, as we decreased the number of cancer cells in xenografts, lung metastasis and then primary tumor growth was impaired in mice injected with the parental SUM149-Luc cells, but not in mice injected with glutamine-independent cells. These observations, particularly from the mice injected with 20,000 or less cells, suggest that Gln-ind cells are more tumorigenic (more incidence and faster appearance of primary tumor) and more metastatic (more incidence of lung metastasis) than the parental cell line.

Inflammatory breast cancer is well known to metastasize to skin. To compare Gln-ind cells with the parental SUM149-Luc cell line for an ability to cause skin metastasis, we chose representative luciferase images in the two groups such that the primary tumor burden in the two groups was comparable (Fig. S6). Although we imaged the mice on different days, we normalized the images for comparison using a common minimum and maximum setting in the Living Image program. In this manner, we found that Gln-ind cells caused more skin metastasis than the parental cells, which is observed as luciferase-positive spots scattered on the body (see spots inside the red rectangles in Fig. S6). We confirmed metastasis to skin by performing *ex vivo* luciferase imaging on dissected skin from two mice at the end of the experiment. This data, showing luciferase positive signal in the skin of a mouse each injected with the parental SUM149-Luc and Gln-ind cells, is presented (Fig. S7). These data along with the lung metastasis data presented above indicate an overall more malignant nature of Gln-ind cells than the parental cell line.

On a separate note, although we had not designed this study to compare metastases to other organs, we analyzed metastases to brain and liver in one mouse each injected with 200,000 SUM149-Luc cells, and 20,000 Gln-ind cells. This data along with luciferase images, tumor weight, and lung metastasis information for these mice is shown (Fig. S8). We found both mice to be positive for metastases in both brain and liver by luciferase activity assay on tissue homogenates, indicating that the xenograft mouse model recapitulates aggressive features of IBC.

### Overexpression of cadherin11 and vimentin in Gln-ind cells

**Figure 15:**
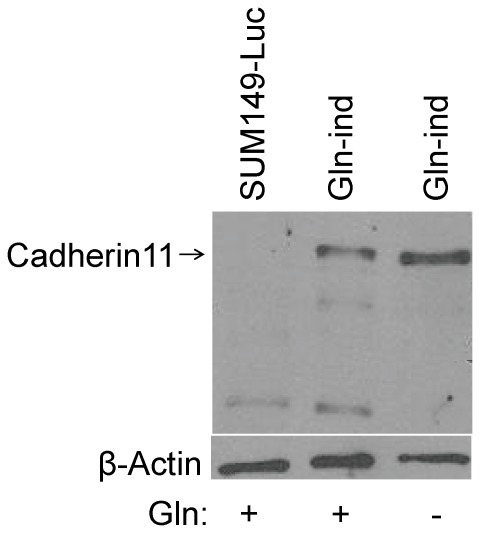
Increased level of cadherin11 in Gln-ind cells. We detected cadherinl1 level by western blotting in parental SUM149-Luc cell line and Gln-ind cell line both growing in a medium with Gln, and Gln-ind cells growing without Gln as indicated at the bottom.

**Figure 16:**
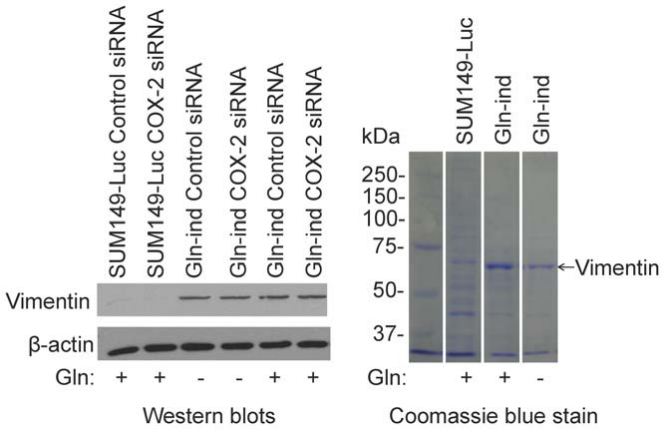
Detection of vimentin in Gln-ind cells. **Left,** We transfected SUM149-Luc and Gln-ind cells with a COX-2 specific siRNA or a negative control siRNA, allowed cells to grow for 3 days with or without Gln as indicated at the bottom, and then subjected them to western blotting to detect vimentin. **Right,** Lysates from parental SUM149-Luc cell line and Gln-ind cells were analyzed by SDS-PAGE followed by Coomassie blue staining. A prominent vimentin-size band, which is present in Gln-ind cells and absent in parental cell line, is indicated by an arrow. Left lane shows prestained molecular weight markers. All samples were run on a single gel; however, we excised some irrelevant lanes that were present between the lanes of interest.

To determine a molecular basis of increased metastasis, we analyzed cadherin11 level in Gln-ind cells. We predicted a high cadherin11 expression from a gene expression profile in COX-2 overexpressing celecoxib-resistant SUM149-CER cell line (B Singh and A Lucci, unpublished data). Cadherin11, also known as mesenchymal cadherin, is expressed in cancer cells undergoing epithelial to mesenchymal transition (EMT). Cadherin11 could be important in cell motility, invasion, homotypic cell-cell interactions, and in cancer cell interaction with stroma [39]. We found by western blot analysis that cadherin11 level is significantly higher in Gln-ind cells than parental SUM149 cells (Fig. 15). Others have shown that EMT correlates with cancer stem cell phenotype [40]. We also analyzed intermediate filament protein vimentin, another important marker of EMT, and found by western blotting and by Coomassie blue staining that vimentin is overexpressed in Gln-ind cells (Fig. 16). We also found that siRNA-mediated COX-2 knockdown in a transient transfection experiment did not affect vimentin level (Fig. 16), as would be expected for a relatively stable protein.

In a separate experiment, we found that, similar to our previous study showing high pro-urokinase plasminogen activator (pro-uPA) expression in COX-2 overexpressing breast cancer cell lines [20], pro-uPA is overexpressed in Gln-ind cells (B Singh, AM Cady, and A Lucci, unpublished data), providing a basis of invasion into basement membrane. These results, along with the results on cellular adaptability, provide a basis of increased tumorigenicity and metastatic potential of Gln-ind cells.

## Discussion

Genetic and epigenetic heterogeneity among cancer cells in a tumor is a major impediment to personalized therapy. Current approaches of personalized therapy, mostly designed to inhibit cell proliferative function of a single protein, may not succeed unless we target adaptability of cancer cells as well. Our study suggests that it is feasible to select highly adaptable rare cancer cells by depriving them of glutamine for an extended period. This approach can be extended to other components in the culture medium, such as glucose, other amino acids, oxygen, etc. Different deprivations will likely select for common elements that are important in adaptable metabolic state, as well as some nutrient-specific elements required for survival and growth under these conditions. Currently, therapeutic approaches are being designed to inhibit cancer metabolism in the bulk of proliferating cancer cells [41], [42]. Investigation of highly adaptable metastatic cancer cells that survive without specific nutrients may teach us about the mechanisms of resistance to metabolism-based and cell proliferation-based therapies and help develop strategies to counter such resistance.

The selection in a medium without glutamine or without glucose would likely favor cells that are able to survive high oxidative stress. Aggressive breast cancer cell lines are characterized by a high level of oxidative stress as indicated by low GSH and low GSSG levels [43]. Since SUM149 is a very aggressive cell line, it is likely to have a relatively high oxidative stress. Lack of nutrients is likely to further increase stress, possibly killing most cells. An alternative reason for the death of most (>99.99%) cells upon glutamine withdrawal could be nutrient sensing response, not directly involving stress response [14]. The rare cells that survive do so either by an ability to tolerate more stress or by other mechanisms for dealing with nutrient unavailability. Our results indicate that Gln-ind cells have even lower levels of GSH and GSSG than the parental cell line (Fig. S1). Our results also provide evidence that oxidative stress in Gln-ind cells may be responsible for COX-2 overexpression and NFκB activation (Figs. 7, S2). There is evidence that stress influences epigenetic state by affecting DNA methylation and histone modifications [44]. It is reasonable that the epigenetic state created in this manner would also allow tolerance to the stress. We favor the possibility that the rare cells able to tolerate extreme stresses including those imposed by metabolic challenges are endowed with a unique epigenetic state.

One way to explain tumor heterogeneity and therapeutic resistance is that a subpopulation of stem-like cancer cells governs these properties. The idea that rare cancer cells resembling stem cells or progenitor cells in the breast drive breast cancer has a widespread support. In this study with an emphasis on clinical translation, we approached the problem of cellular heterogeneity by selecting for metabolically plastic aggressive cancer cells. We believe that the cellular adaptability may be an important feature of stemness. In this regard, a recent study found that glioma stem cells, but not “differentiated” glioma cells, possess a flexible metabolic state that allows them to use multiple metabolic pathways for producing energy [45]. In the current and future studies, it will be our high priority to determine whether we can eradicate Gln-ind cells with therapies being developed to target cancer stem cells.

How does our method of enriching metastatic cancer cells compare with other function-based *in vitro* selection methods? One prominent method, which has been used in several studies with cancer cell lines from different organ sites including breast, involves selection of anchorage-independent cells in soft agar or hard agar. The method selects for invasive cells that are metastatic in xenograft mouse models [46]–[50]. Importantly, highly anchorage-independent cells selected in this manner are metastatic not only in immunocompromised nude mice, but also in immunocompetent syngeneic mice [46], [47]. Significantly, Gln-ind cells are more anchorage-independent than parental cells (Fig. 10B). It will be useful to find out in the future whether metastatic cancer cells selected on hard agar are more metabolically adaptable as well. It will also be important to learn whether Gln-ind cells are metastatic in immunocompetent mice. This issue can be addressed by using the Gln-ind cells we have selected from mouse breast cancer cell lines 4T1, 4T07, and 168FARN (Fig. 2). The advantages in our selection as compared to growth in hard agar are that our selection is well defined and easy to perform, and such selection is likely to occur in the body. Our method has the likelihood of being applicable to most tumor types and subtypes, and capable of selecting a large spectrum of cellular heterogeneity. To conclude, both methods have unique strengths that make them useful. Although the two selection methods are different at the surface, both may be able to select adaptable cells, one by virtue of metabolic plasticity, and the other one by virtue of structural plasticity.

The question at this time is whether the *in vitro* assays will be useful in discovering safe and effective therapies to prevent and cure metastasis. Obviously it would be better to attack the problem before micrometastases progress to clinical metastasis. Given a high failure rate of new therapies in the clinic, it is most important that we have superior preclinical models, and that candidate therapies pass more rigorous tests of efficacy before they are tested in patients. Then we will be in a better position to attack the disease that has often “evolved” in the body for decades. We believe superior *in vitro* testing will play a significant role in the development of combination therapies that will be required to combat aggressive cancers.

## Materials and Methods

### Ethics statement

The studies with nude mice were performed under an animal protocol (ACUF Protocol # 12-10-11431 entitled “Metabolic plasticity in breast cancer metastasis”, Principal Investigator- Anthony Lucci) approved by the University of Texas MD Anderson Cancer Center Institutional Animal Care and Use Committee.

### Cell lines and culture

MCF10A and MCF10A-COX2 (COX-2-transfected) cell lines [25] were maintained in DMEM/F12 medium supplemented with 5% heat-inactivated horse serum (Invitrogen, Carlsbad, CA), 10 µg/ml of insulin, 20 ng/ml of epidermal growth factor, 0.1 µg/ml of cholera toxin, 0.5 µg/ml of hydrocortisone, 100 units/ml of penicillin, and 100 µg/ml of streptomycin. The MCF7, MDA-MB-231, and MDA-MB-231-BSC60 cell lines were grown in RPMI 1640 medium supplemented with 10% fetal bovine serum (FBS), 100 U/ml of penicillin, and 100 µg/ml of streptomycin. MDA-MB-231-BSC60 is a metastatic variant of MDA-MB-231 isolated in our laboratory after two rounds of selection in female nude mice by cardiac ventricle inoculation followed by cell culture from bone metastases [21]. The SUM149 and SUM190 inflammatory breast cancer cell lines, originally obtained from Stephen Ethier (Barbara Ann Karmanos Cancer Institute, Detroit, MI, USA), were grown in Ham's F-12 medium supplemented with 5% FBS, 5 µg/ml of insulin, 1 µg/mL of hydrocortisone, 100 U/ml of penicillin, and 100 µg/ml of streptomycin in a humidified 5% CO_2_ atmosphere. We have previously described SUM149-Luc, a luciferase-transfected cell line [37], and SUM149-CER, a celecoxib-resistant COX-2 overexpressing cell line [30]. SUM149-FP is another cell line recently developed in our laboratory by first culturing from a SUM149 xenograft in a female nude mouse and then culturing from a fat pad xenograft. 4T1, 4T07, and 168FARN mouse breast cancer cell lines with different metastatic potential, originally isolated in the laboratory of Fred Miller [51], were obtained from Ralph Arlinghaus (MD Anderson Cancer Center) and grown in DMEM/F-12 medium supplemented with 10% FBS.

### Assay of glutamine dependence of cell lines

Initially, we surveyed breast cancer cell lines for their relative dependence on glutamine (Gln) for growth. For this purpose, we plated half a million cells in 10-cm dishes. The next day, we removed the medium, rinsed the dishes twice with phosphate-buffered saline, and added new medium containing dialyzed FBS (Invitrogen) and lacking Gln. We followed the cultures to determine the effects of long-term deprivation of Gln. If a culture grew with little effect of Gln deprivation, e.g., the MCF10A cell line, we passaged it at least 3 times in Gln-free medium to confirm that the culture can grow without Gln. On the other hand, if Gln withdrawal in the initial dish resulted in near-complete inhibition, e.g., in the highly aggressive SUM149 cell line, we followed these dishes for 3–4 weeks to determine if some variants would survive and produce colonies.

### Selection and culture of Gln-ind variants

The main purpose of this study was to investigate rare Gln-ind variants in aggressive breast cancer cell lines. We noticed in the initial experiments that the cell lines that were cultured in Ham's F-12 medium yielded healthier colonies of Gln-ind cells, and the Gln-ind cells proliferated well in this medium. Therefore, we utilized both Ham's F-12 medium and the culture medium in which a particular cell line is usually cultured to select Gln-ind variants. In this manner, we found that Ham's F-12 medium is superior to other media for selecting and culturing Gln-ind cells. To make the selection highly robust, we waited for at least 3–4 weeks, until the Gln-addicted cells were completely eliminated. When the colonies grew to a size easily visible with the naked eye, we counted the number of colonies and began passaging them for long-term culture. Most Gln-ind variants selected in this manner could be cultured long-term in Gln-free medium. However, Gln-ind variants isolated from some cell lines required Gln for long-term culture.

### COX-2 knockdown with siRNA

We performed COX-2 knockdown in the SUM149-CER cell line [30] using COX-2-specific Silencer Select siRNA s11473 and a Silencer Select negative control #1 siRNA (both from Applied Biosystems, Foster City, CA), along with siPORT NeoFX transfection agent (Applied Biosystems), according to the manufacturer's instructions. We performed COX-2 knockdown in Gln-ind cells similarly, except that we used culture medium lacking Gln during the transfection.

### Western immunoblotting

We separated proteins by sodium dodecyl sulfate polyacrylamide gel electrophoresis (SDS-PAGE) and detected various proteins by western blotting, as described previously [18] using the ECL Advance western blot detection reagents (GE Healthcare Biosciences, Piscataway, NJ). The following primary antibodies were used for detection: anti-COX-2 monoclonal antibody (Cayman Chemical, Ann Arbor, MI), anti-GLS (Abcam, Cambridge, MA), anti-Myc, anti-LDH A, anti-hexokinase II, anti-GAPDH, anti-cadherin11, and anti-vimentin antibodies (Cell Signaling, Danvers, MA). The filters were stripped by incubating the membrane in 0.5% Triton X-100 and were re-probed with a monoclonal β-actin antibody (Sigma-Aldrich, St. Louis, MO) or with Hsp90 antibody (Cell Signaling), which served as gel-loading controls. We performed each western blot at least 3 times. We quantified the protein bands on x-ray films by using the ImageJ image processing program (National Institutes of Health).

### Adaptability assays

To determine the adaptability of the Gln-ind cells, we developed two assays: one for adaptability to face metabolic challenges and the other one to face a lack of growth factors. To determine adaptability of the metabolic state of Gln-ind cells, we deprived them of glucose for 28 days and assessed whether a higher number of cells survived compared with those surviving glucose deprivation in the parental cell line. Cancer cells are highly addicted to glucose, so the majority of them are expected to die in the absence of glucose. The basis of the assay is that the survival of rare cells under extreme glucose deficiency for an extended period is likely to select for cells with a highly adaptable metabolic state. We plated a half million cells on a 10-cm dish, switched them to glucose-free medium (custom medium from (AthenaES, Baltimore, MD) for 28 days, and then switched them back to complete medium for 2 weeks until the surviving cells yielded colonies. We stained the colonies with crystal violet and photographed them.

Factors present in serum regulate cell, which also involves instructing a cell to proliferate. The rationale for our second assay is that highly adaptable rare cancer cells that drive the disease must be relatively less dependent on extrinsic factors as compared to the majority of cancer cells. In this assay, we plated a half million cells on a 10-cm dish and switched them to medium without serum on a permanent basis, thus allowing sufficient time for the selection of rare, highly adaptable cells that would survive and grow into colonies. We stained the colonies with crystal violet and photographed them.

### Soft-agar colony-forming assay

We plated 12,000 cells per well in a 6-well dish in 0.35% low-melt agarose (Fisher Scientific, Pittsburgh, PA) in 1 mL of culture medium containing glutamine and dialyzed FBS, which was layered on the top of a 1-ml 0.5% agarose layer in the same medium. We replenished the medium by adding 0.2 ml of medium per well on a weekly basis. After 23 days, we stained the colonies with crystal violet and photographed them.

### Assays for resistance to chemotherapeutic drugs and celecoxib

We plated one million cells per 10 cm dish in duplicate in culture medium with glutamine. After 24 hours, we added doxorubicin (0–400 nM) or paclitaxel (0–10 nM) dissolved in DMSO. We continued drug treatment up to seven days while monitoring drug response under microscope. Then we removed the drug-containing medium, washed twice with PBS, and incubated in the Gln-containing medium without drug for several days until colonies visible to the naked eye appeared. Colonies were stained with crystal violet and photographed. We determined resistance to celecoxib in a similar manner by treating the cells with 50 µM celecoxib for 5 days and then allowing the rare most resistant cells to grow into colonies.

### Xenograft experiments

We anesthetized 20 female athymic nude mice (Harlan, Indianapolis, IN) 44 days old with isoflurane inhalation. We injected Gln-ind or parental SUM149-Luc cells in amounts of 2 million, 0.2 million, 20,000, 2,000, or 200 cells each in duplicate mice orthotopically into the upper left mammary fat pad. For each injection, we suspended cancer cells in 0.05 ml of serum-free medium and mixed them with an equal volume of Matrigel (Fisher Scientific). Starting 14 days from the date the cancer cells were injected, we used whole-body luciferase imaging (Xenogen IVIS System 200; Caliper Life Sciences, Hopkinton, MA) to detect primary tumors and skin metastases. Each mouse was given 1 mg potassium salt of D-luciferin in 0.1 ml phosphate-buffered saline by intraperitoneal injection and then anesthetized with isoflurane. Starting 5 min after the D-luciferin injection, bioluminescent images were collected for 1 min each in the ventral position. Generally, five mice were imaged at a time. The levels of light emitted from the bioluminescent tumor cells were detected by the IVIS camera system, integrated, digitized, and displayed. We refer to tumor in the fat pad as the primary tumor for clarity, although it was not possible to distinguish primary tumors from local metastases. At the end of the experiment, we euthanized the mice by CO_2_ inhalation and resected and weighed the tumors. To quantify lung metastases, we harvested lungs in 1 ml cell lysis buffer (25 mM Tris–phosphate, pH 7.8, 2 mM dithiothreitol, 2 mM 1,2-diaminocyclohexane-N,N,N′,N′-tetraacetic acid, 10% glycerol, 1% Triton X-100), prepared the lysates by chopping the lungs into small pieces with a scalpel followed by dounce homogenization, and assayed relative luciferase activity with a kit (Promega) and a luminometer.

## Supporting Information

Figure S1 **A low level of glutathione in Gln-ind cells.** We prepared cell lysates from SUM149-Luc cell line (parental) and Gln-ind cell line, both growing in a medium with Gln, and measured reduced glutathione (GSH) and oxidized glutathione (GSSG) with a kit from BioVision (Milpitas, CA). The GSH and GSSG levels represent an average of 3 measurements normalized to an equal protein basis.
(TIFF)

Figure S2 **A reduction in COX-2 level upon treatment with BAY-11-7082.** We exposed parental SUM149-Luc and Gln-ind cell lines both growing in a medium with glutamine (top and middle panel), and Gln-ind cells growing without Gln (bottom panel) to indicated concentrations of BAY-11-7082 or to DMSO solvent alone for 24 hours before subjecting them to western blotting using equal volumes of cell lysates.
(TIFF)

Figure S3 **Enrichment of Gln-ind phenotype upon prior selection of chemotherapeutic resistance.** Parental SUM149-Luc cells were treated with DMSO solvent alone or with 200 nM doxorubicin for 3 days, allowed to recover in a drug-free medium for 17 days, and then trypsinized and plated in glutamine-free medium for 32 days before staining (top). Similarly, parental SUM149-Luc cells were treated with DMSO solvent alone or with 5 nM paclitaxel for 3 days, allowed to recover in a drug-free medium for 28 days, and then trypsinized and plated in glutamine-free medium for 34 days before staining (bottom).
(TIFF)

Figure S4 **Increased glutamine independence phenotype in celecoxib-resistant cells.** Parental SUM149-Luc cell line was treated with DMSO solvent or with 10 µM celecoxib for 7 days. Celecoxib-resistant cells were allowed to recover in drug-free medium for 3 days, and then trypsinized and plated in glutamine-free medium at 0.5 million cells per 10 cm dish. Gln-ind colonies were stained with crystal violet after 34 days.
(TIFF)

Figure S5 **Gln-ind cells were more tumorigenic and more metastatic in nude mice than the parental cell line.** Cells (200 to 2 million) were injected into thoracic fat pads of 44-day-old nude mice (decreasing cell number from left to right) in duplicate. Luciferase images collected at day 34 after injecting cancer cells show higher tumor growth (compare luciferase signal around fat pad) and skin metastasis (compare signal in dots away from the injection site) in mice injected with Gln-ind cells (bottom) than the parental SUM149 cell line (top). Empty slots on the left correspond to the mice that needed to be sacrificed because of high tumor burden.
(TIFF)

Figure S6 **Increased skin metastasis in nude mice injected with Gln-ind cells.** Luciferase images of mice injected with indicated cell type and cell number were collected at different days. Images were chosen to have approximately similar luciferase signal in primary tumors in mice injected with parental SUM149 (top) and Gln-ind cells (bottom). Skin metastases are indicated as luciferase signals in red rectangles.
(TIFF)

Figure S7 ***Ex vivo*** **imaging to confirm skin metastasis.** Skin away from primary tumor was dissected at day 25 from the mice injected with 2 million parental SUM149-Luc cells (top) or Gln-ind cells (bottom). The skin was placed in a petri dish containing 0.5 mg D-luciferin in 10 ml PBS for 5 min, and imaged with the Xenogen IVIS System 200.
(TIFF)

Figure S8 **Detection of metastases to brain and liver.** Luciferase images of two mice injected with indicated cell type and cell number were collected at the indicated days. The mouse injected with the parental SUM149-Luc cells was sacrificed at day 67 (5 days after luciferase imaging), and the mouse injected with Gln-ind cells was sacrificed at day 50 (2 days after luciferase imaging). Tumors were weighed, and tissues were homogenized (lungs and brain each in 1 ml, and liver in 1.5 ml) and luciferase activity assayed as described for lung metastasis in Materials and Methods. Total luciferase activity in tissues is shown as relative luminescence units.
(TIFF)
