# Supplementary figures and images for: Selection of Metastatic Breast Cancer Cells Based on Adaptability of Their Metabolic State
Source: PLoS One. 2012 May 3;7(5):e36510. doi: 10.1371/journal.pone.0036510 (PMC3343010; doi:10.1371/journal.pone.0036510)

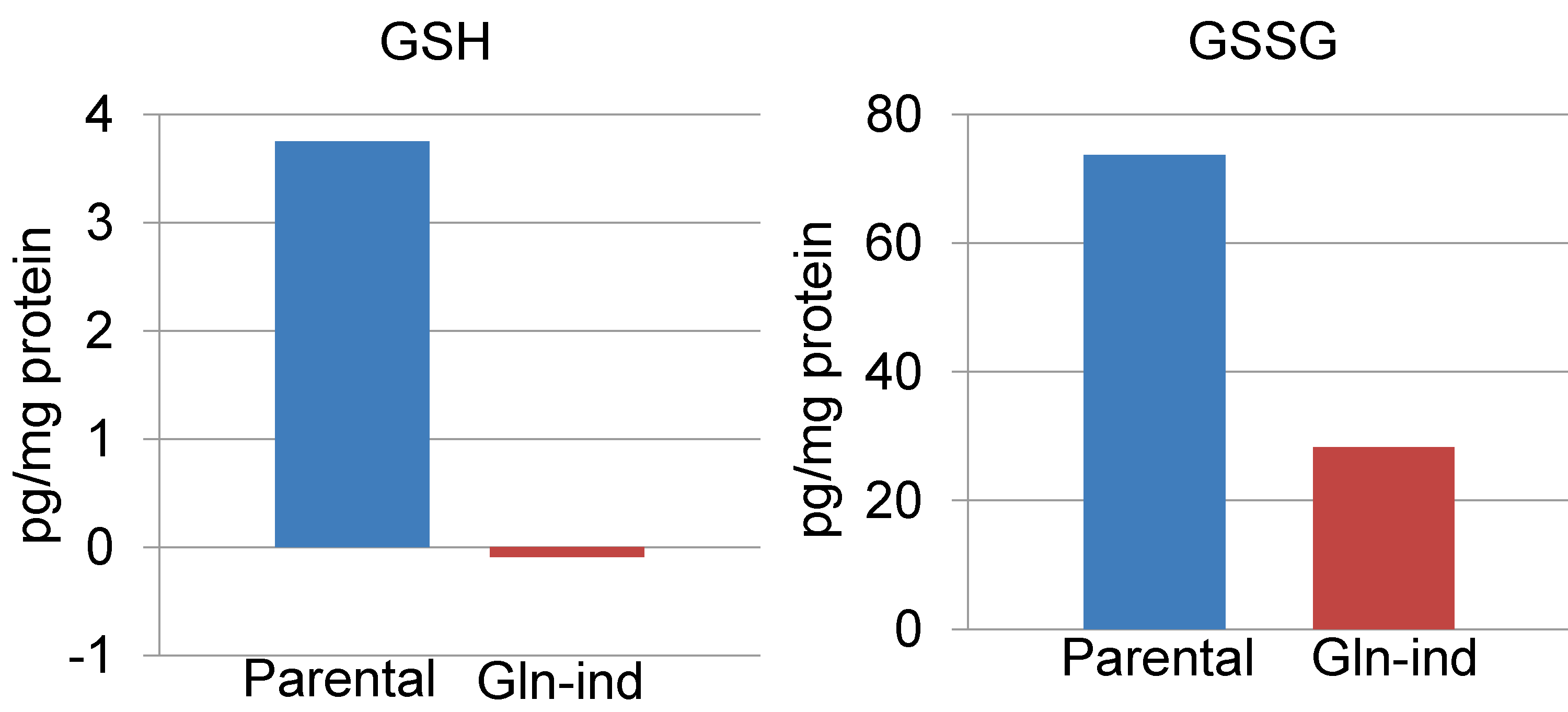

Supplement: Figure S1 — A low level of glutathione in Gln-ind cells. We prepared cell lysates from SUM149-Luc cell line (parental) and Gln-ind cell line, both growing in a medium with Gln, and measured reduced glutathione (GSH) and oxidized glutathione (GSSG) with a kit from BioVision (Milpitas, CA). The GSH and GSSG levels represent an average of 3 measurements normalized to an equal protein basis. (TIFF) [file pone.0036510.s001.tiff]

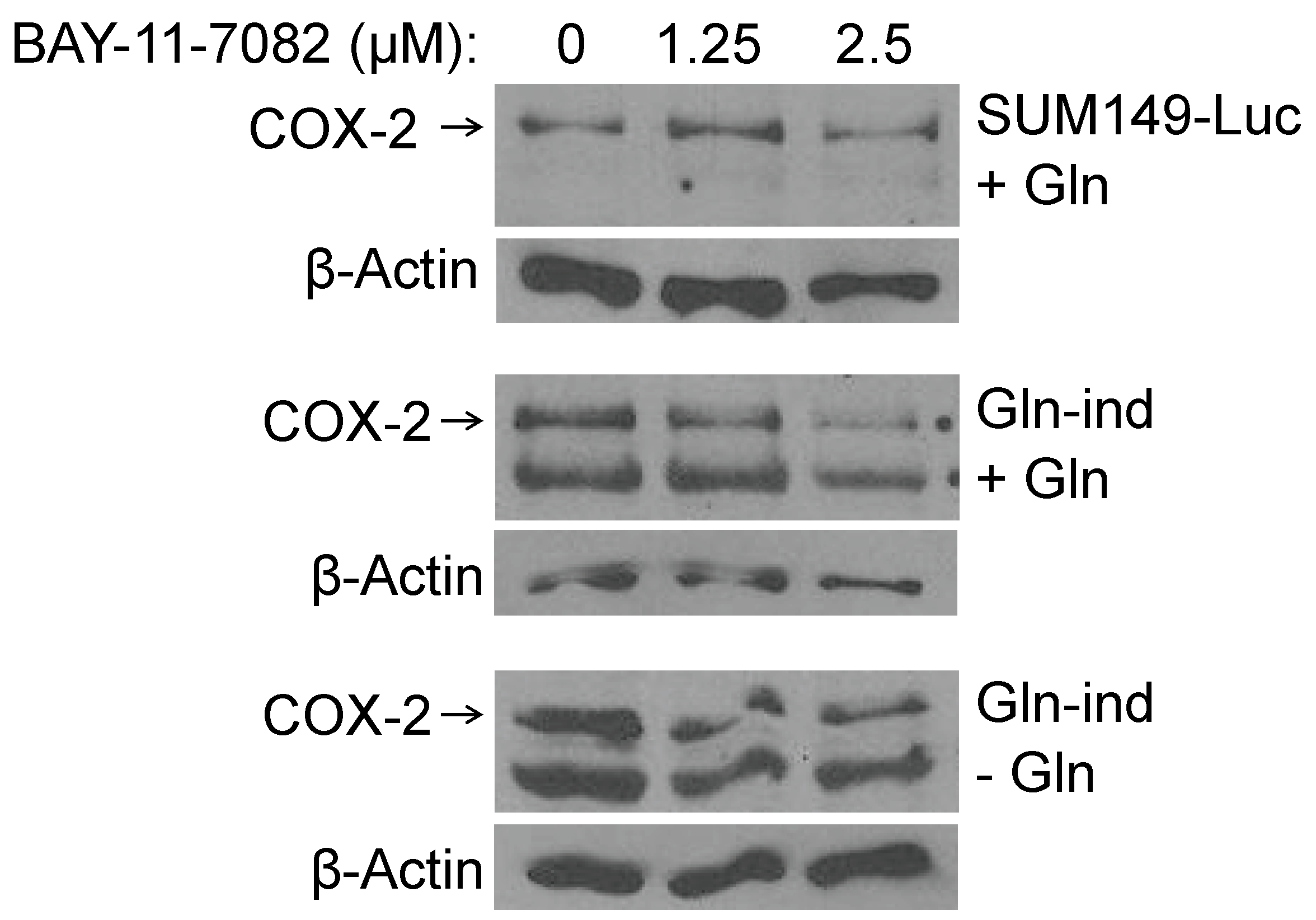

Supplement: Figure S2 — A reduction in COX-2 level upon treatment with BAY-11-7082. We exposed parental SUM149-Luc and Gln-ind cell lines both growing in a medium with glutamine (top and middle panel), and Gln-ind cells growing without Gln (bottom panel) to indicated concentrations of BAY-11-7082 or to DMSO solvent alone for 24 hours before subjecting them to western blotting using equal volumes of cell lysates. (TIFF) [file pone.0036510.s002.tiff]

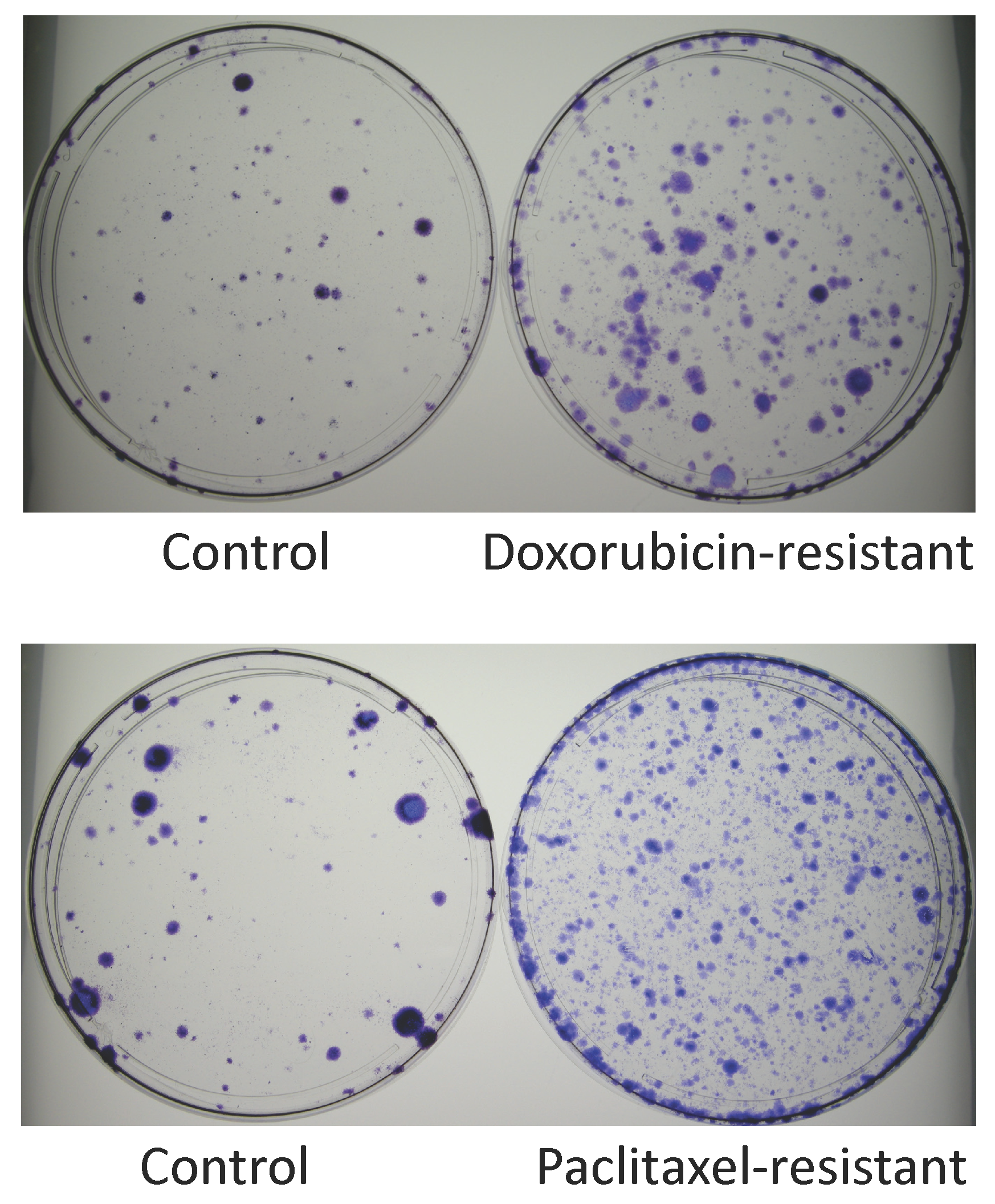

Supplement: Figure S3 — Enrichment of Gln-ind phenotype upon prior selection of chemotherapeutic resistance. Parental SUM149-Luc cells were treated with DMSO solvent alone or with 200 nM doxorubicin for 3 days, allowed to recover in a drug-free medium for 17 days, and then trypsinized and plated in glutamine-free medium for 32 days before staining (top). Similarly, parental SUM149-Luc cells were treated with DMSO solvent alone or with 5 nM paclitaxel for 3 days, allowed to recover in a drug-free medium for 28 days, and then trypsinized and plated in glutamine-free medium for 34 days before staining (bottom). (TIFF) [file pone.0036510.s003.tiff]

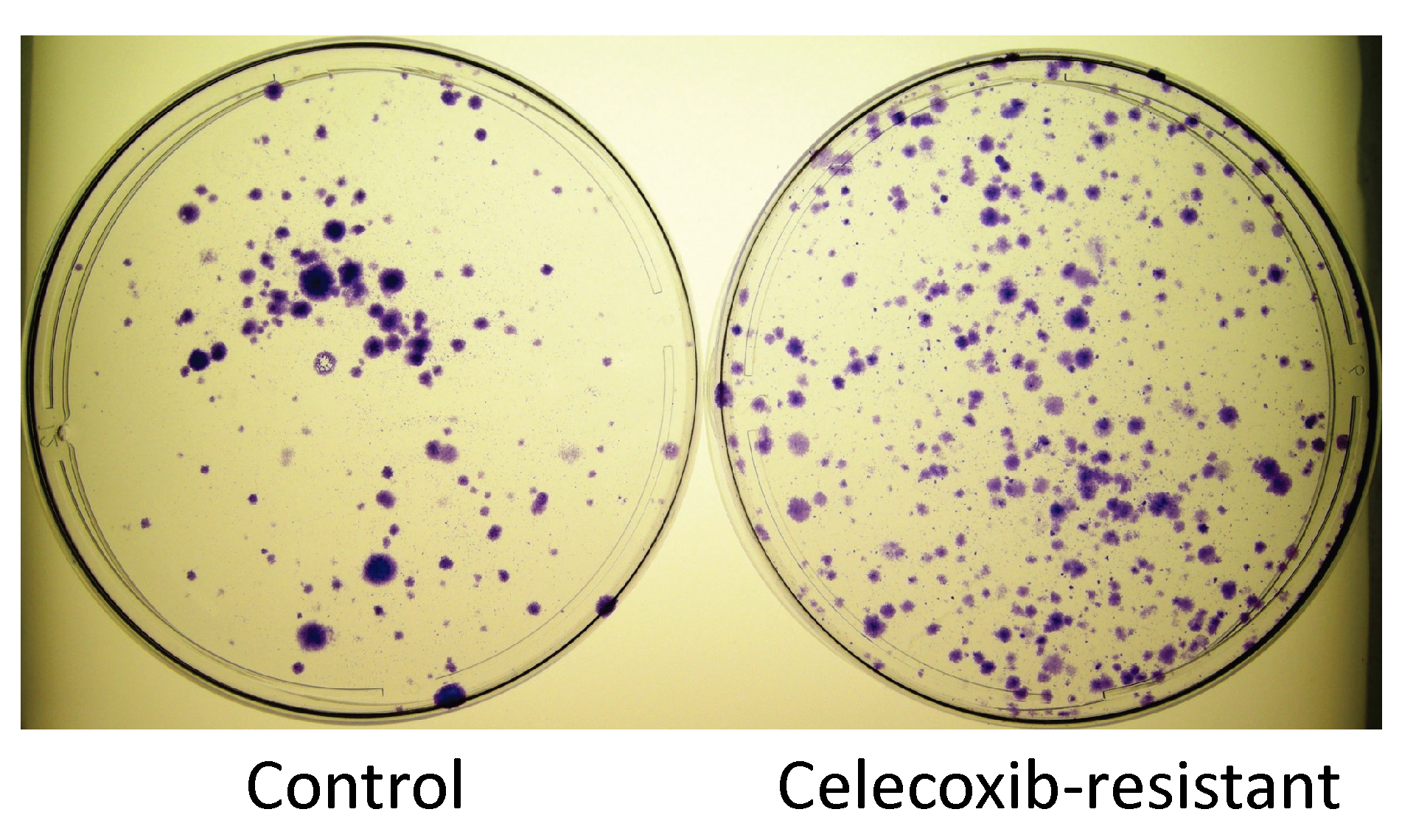

Supplement: Figure S4 — Increased glutamine independence phenotype in celecoxib-resistant cells. Parental SUM149-Luc cell line was treated with DMSO solvent or with 10 µM celecoxib for 7 days. Celecoxib-resistant cells were allowed to recover in drug-free medium for 3 days, and then trypsinized and plated in glutamine-free medium at 0.5 million cells per 10 cm dish. Gln-ind colonies were stained with crystal violet after 34 days. (TIFF) [file pone.0036510.s004.tiff]

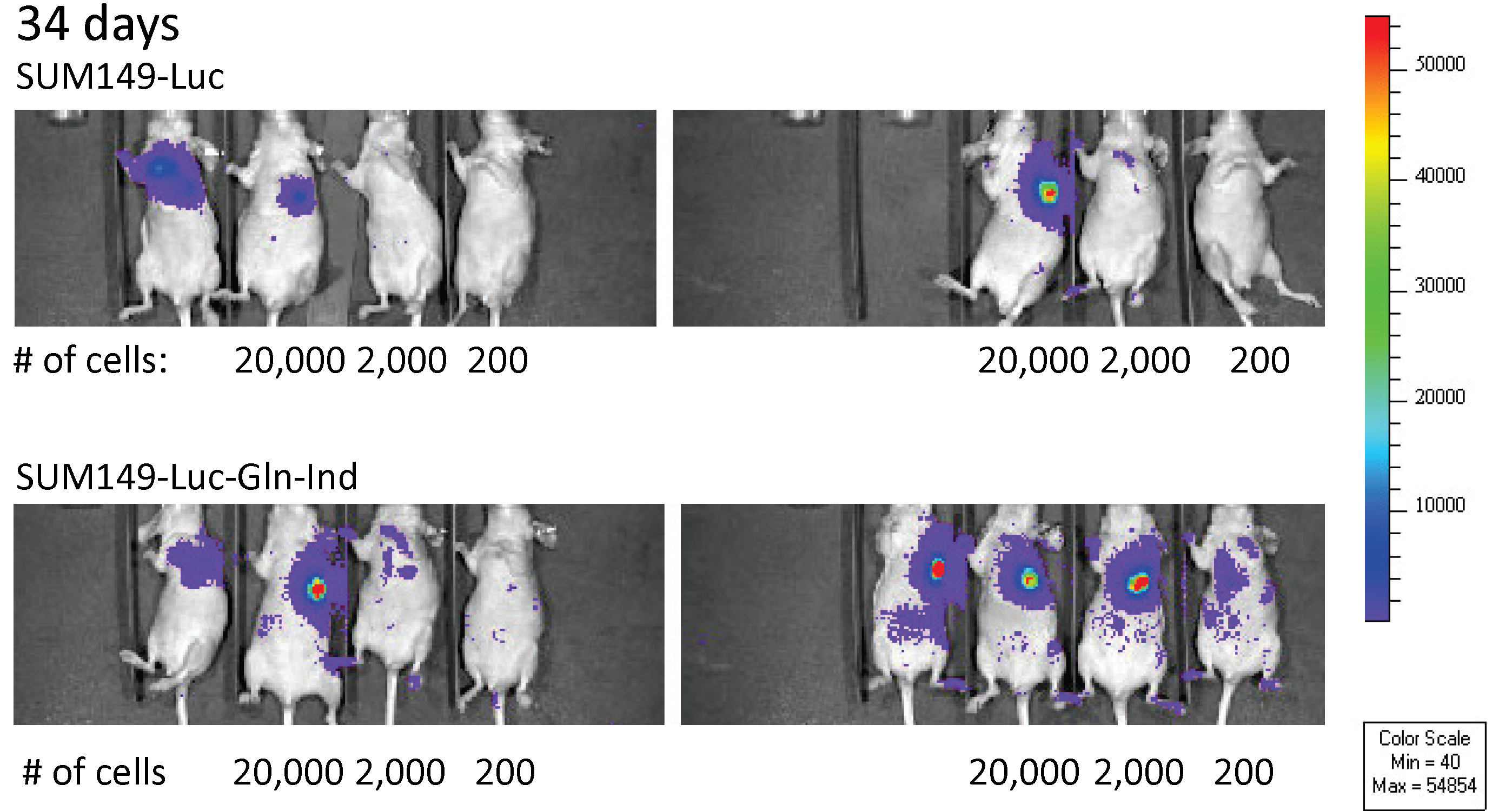

Supplement: Figure S5 — Gln-ind cells were more tumorigenic and more metastatic in nude mice than the parental cell line. Cells (200 to 2 million) were injected into thoracic fat pads of 44-day-old nude mice (decreasing cell number from left to right) in duplicate. Luciferase images collected at day 34 after injecting cancer cells show higher tumor growth (compare luciferase signal around fat pad) and skin metastasis (compare signal in dots away from the injection site) in mice injected with Gln-ind cells (bottom) than the parental SUM149 cell line (top). Empty slots on the left correspond to the mice that needed to be sacrificed because of high tumor burden. (TIFF) [file pone.0036510.s005.tiff]

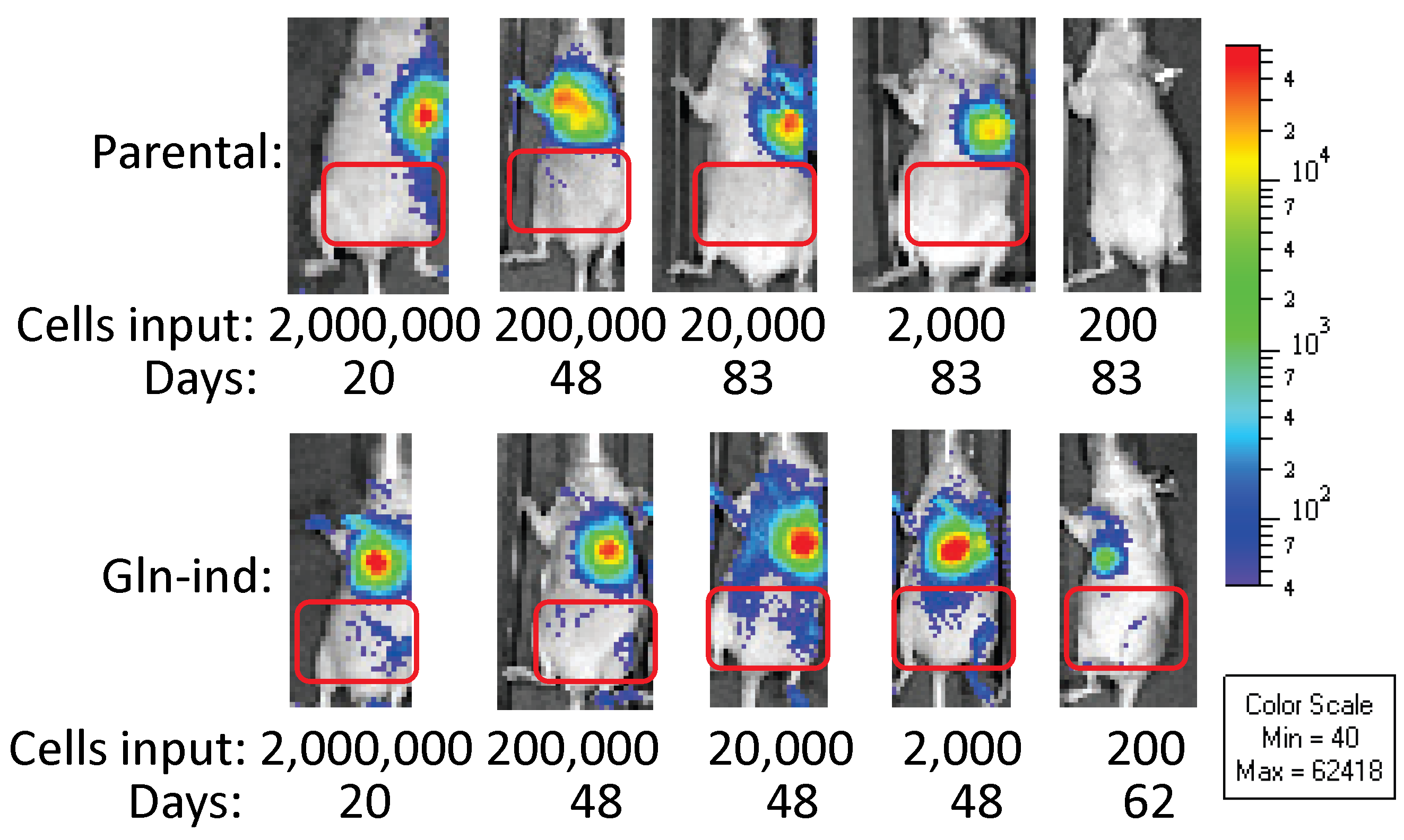

Supplement: Figure S6 — Increased skin metastasis in nude mice injected with Gln-ind cells. Luciferase images of mice injected with indicated cell type and cell number were collected at different days. Images were chosen to have approximately similar luciferase signal in primary tumors in mice injected with parental SUM149 (top) and Gln-ind cells (bottom). Skin metastases are indicated as luciferase signals in red rectangles. (TIFF) [file pone.0036510.s006.tiff]

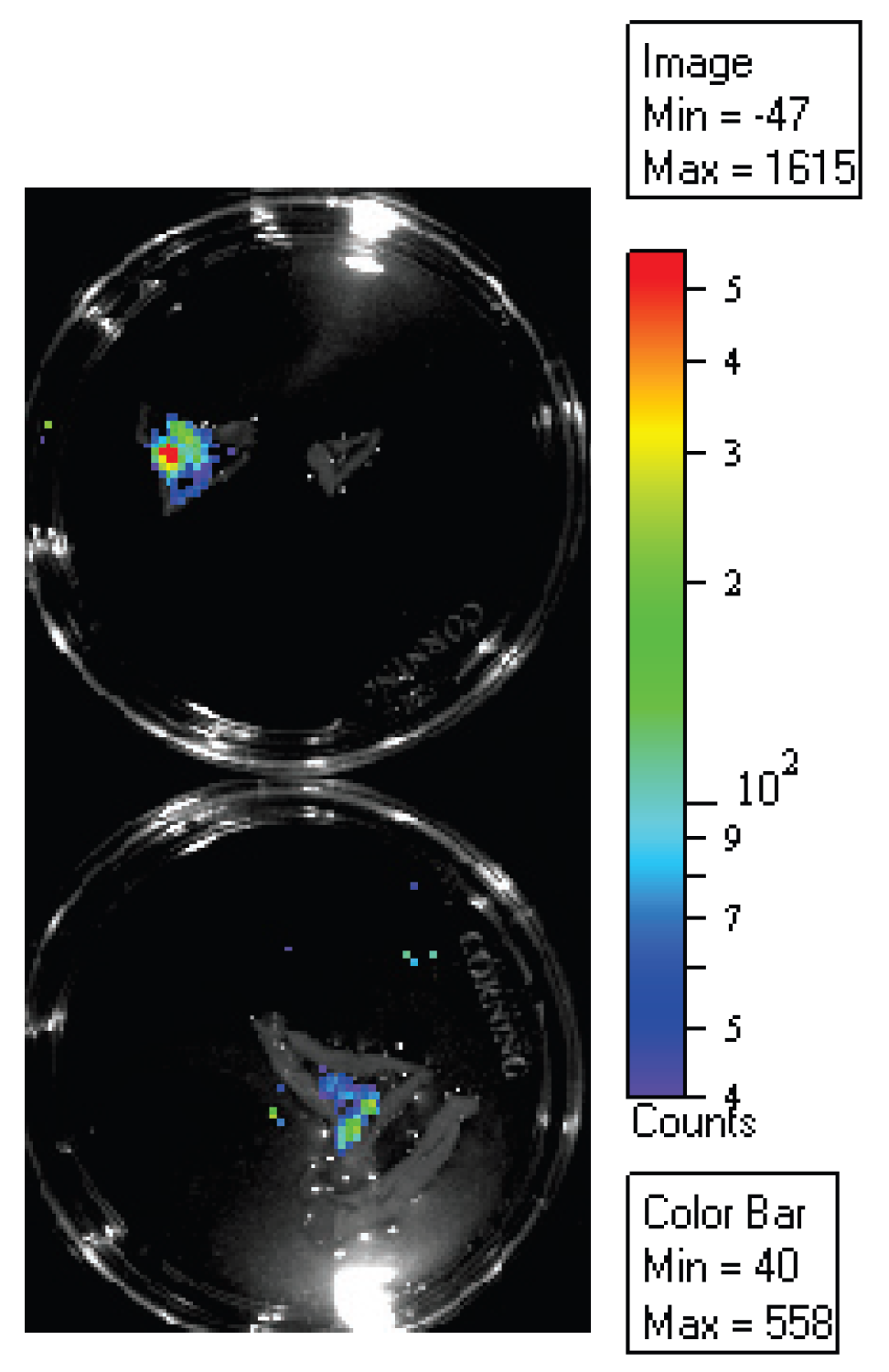

Supplement: Figure S7 — Ex vivo imaging to confirm skin metastasis. Skin away from primary tumor was dissected at day 25 from the mice injected with 2 million parental SUM149-Luc cells (top) or Gln-ind cells (bottom). The skin was placed in a petri dish containing 0.5 mg D-luciferin in 10 ml PBS for 5 min, and imaged with the Xenogen IVIS System 200. (TIFF) [file pone.0036510.s007.tiff]

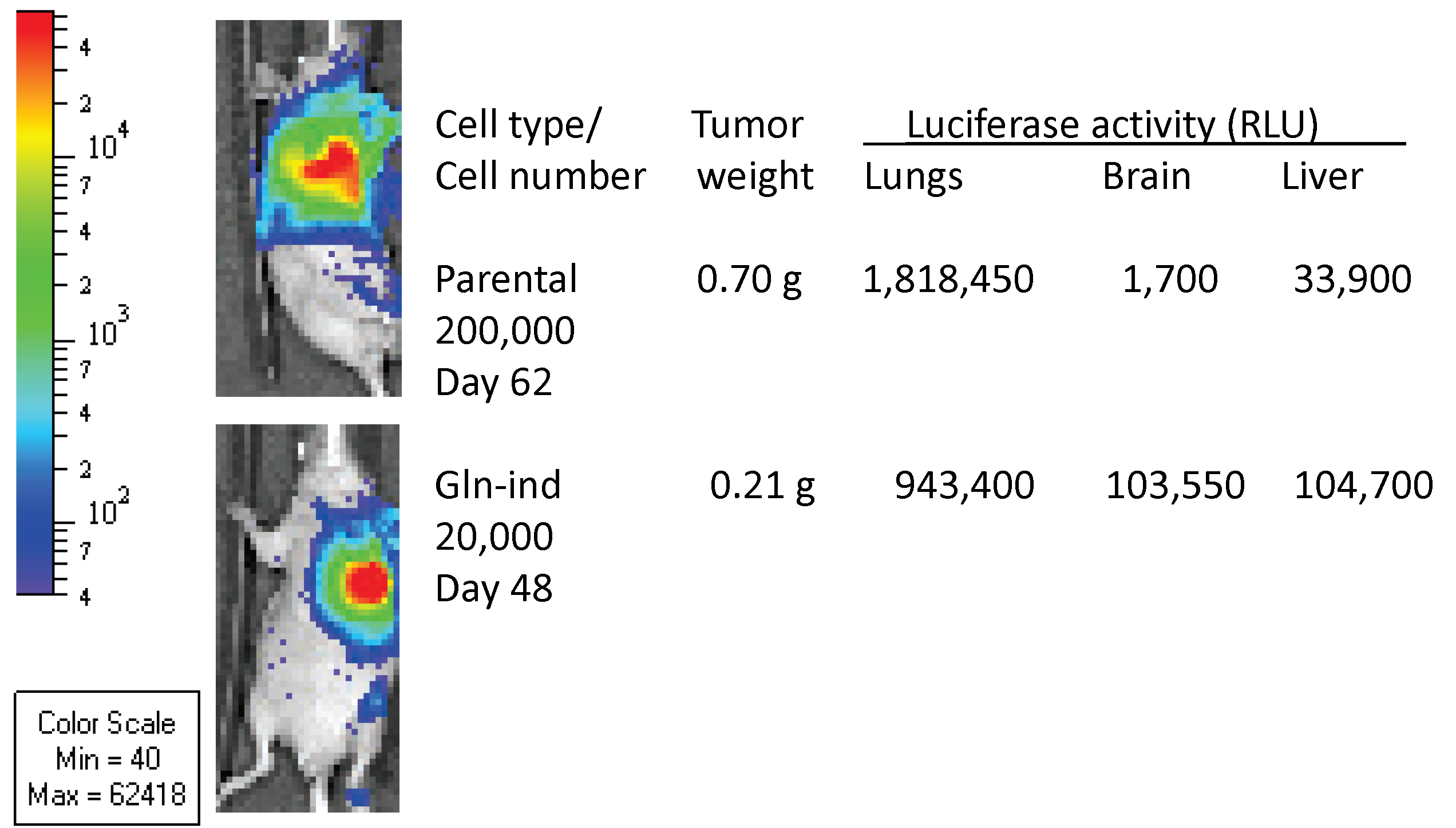

Supplement: Figure S8 — Detection of metastases to brain and liver. Luciferase images of two mice injected with indicated cell type and cell number were collected at the indicated days. The mouse injected with the parental SUM149-Luc cells was sacrificed at day 67 (5 days after luciferase imaging), and the mouse injected with Gln-ind cells was sacrificed at day 50 (2 days after luciferase imaging). Tumors were weighed, and tissues were homogenized (lungs and brain each in 1 ml, and liver in 1.5 ml) and luciferase activity assayed as described for lung metastasis in Materials and Methods. Total luciferase activity in tissues is shown as relative luminescence units. (TIFF) [file pone.0036510.s008.tiff]
